# Is early measles vaccination associated with stronger survival benefits than later measles vaccination?

**DOI:** 10.1186/s12889-018-5866-y

**Published:** 2018-08-07

**Authors:** Jesper Sloth Hansen, Sanne Marie Thysen, Amabelia Rodrigues, Cesario Martins, Ane Bærent Fisker

**Affiliations:** 1 Research Center for Vitamins and Vaccines (CVIVA), Bandim Health Project, Statens Serum Institut, Copenhagen, Denmark; 2 Bandim Health Project, INDEPTH Network, Bissau, Guinea-Bissau; 3 GloHAU, Centre for Global Health, Department of Public Health, Aarhus University, Aarhus, Denmark; 4 OPEN, Odense Patient data Explorative Network, Odense University Hospital/Institute of Clinical Research, University of Southern Denmark, Odense, Denmark

**Keywords:** Non-specific/heterologous effects of vaccines, Measles vaccine, Childhood mortality

## Abstract

**Background:**

Measles vaccine (MV) may protect against non-measles mortality. We tested whether survival depended on age of measles vaccination.

**Methods:**

Bandim Health Project follows children under 5 years of age through a Health and Demographic Surveillance System in rural Guinea-Bissau. Children aged 6–36 months with a vaccination card inspected were followed to the next visit or for a maximum of 6 months. In Cox proportional-hazards models adjusted for age and village cluster, we compared the survival of children vaccinated with MV early (< 9 months), as recommended (9–11 months) or late (> 12+ months) with the survival of measles-unvaccinated children. Among measles-vaccinated children, we modelled the effect of age at measles vaccination linearly to assess mortality changes per month increase in vaccination age.

**Results:**

From 1999 to 2006, 14,813 children (31,725 observations) were included. Children vaccinated with MV had a Hazard Ratio (HR) of 0.76 (95% CI: 0.63–0.91) compared with measles-unvaccinated children; censoring measles deaths did not change the results (HR = 0.79 (0.65–0.95)). For early MV the HR was 0.68 (0.53–0.87), for MV as recommended the HR was 0.77 (0.62–0.96) and for late MV the HR was 0.86 (0.67–1.11)*.* Limiting the analysis to measles-vaccinated children, age at measles vaccination was associated with a 2.6% (0.4–5.1%) increase in mortality per month increase in vaccination age.

**Conclusion:**

Early MV was associated with a large survival advantage. The current policy to increase vaccination age, when measles control improves, may not optimize the impact of MV on child survival.

**Electronic supplementary material:**

The online version of this article (10.1186/s12889-018-5866-y) contains supplementary material, which is available to authorized users.

## Background

Vaccination policies are based on the assumption that a vaccine only protects against the targeted disease. New knowledge indicates that this perception should be revised: Vaccines may affect the overall mortality and morbidity through training of the immune system. In other words, vaccines seem to have non-specific effects (NSEs) altering susceptibility to non-targeted infections [1].

WHO recently reviewed the evidence for NSEs of the Bacillus Calmette-Guérin vaccine (BCG), diphtheria-tetanus-pertussis vaccine (DTP) and measles vaccine (MV) [2]. For MV, the review concluded that the evidence was consistent with beneficial NSEs, which were stronger for girls than for boys [3]. Current vaccination policies do not take the NSEs into account. WHO recommends MV at 9 months of age in countries with a high burden of measles [4]. In areas where measles infection is controlled, MV is recommended at 12 months of age. Measles vaccination in the presence of maternal measles antibodies (MatAb) reduces the antibody response [5, 6] and delaying vaccination until MatAb have waned optimizes seroconversion [4]. Hence, the recommendation of MV at 9 months of age in low-income countries is a compromise between ensuring protection against measles infection early in life and minimizing interference from MatAb [4].

In a randomized trial from Guinea-Bissau, an early dose of MV at 4.5 months in addition to the routine MV at 9 months of age compared with the standard dose of MV at 9 months reduced mortality between 4.5 and 36 months of age (hazard ratio (HR) 0.70 (95% CI: 0.52–0.94)) [7]. In a subgroup of children for whom MatAb were assessed before vaccination at 4.5 months, the HR between 4.5 and 5 years of age was 0.29 (0.09–0.91) for children with MatAb compared with children without MatAb at time of measles vaccination [8]. Therefore vaccinating earlier and providing MV in the presence of MatAb may modulate the immune system in a beneficial way resulting in better child survival [8, 9]. This is supported by a recent review indicating better survival for children vaccinated before 12 months of age than for children vaccinated after 12 months of age [10].

Though the WHO-recommended age of MV is 9 months, many children in Guinea-Bissau receive MV before that age. We evaluated, whether MV given before 9 months of age was associated with stronger survival advantage than MV given after 9 or 12 months of age.

## Methods

### Setting and study population

Bandim Health Project (BHP) has conducted research based on a Health and Demographic Surveillance System (HDSS) in Guinea-Bissau for 39 years. The rural HDSS was established in 1990, covering 20 clusters of 100 women in each of the five most populous regions in Guinea-Bissau. Women of fertile age and their children are followed through home visits in the villages every 6 months. At all visits, pregnancies are registered and information on vital status, vaccination status and nutritional status is recorded for children up to 5 years of age. For children who died since the previous visits, the respondent is asked to state the date and cause of death. Furthermore, we register all new women and note their ethnicity, years of schooling and age.

Data for the present study were collected between January 1st 1999, just after a civil war, and May 15th 2006, when a national MV campaign started [11]. Children aged 6 to 36 months of age were eligible for the study. The vaccination program in Guinea-Bissau was BCG and oral polio vaccine (OPV) at birth and three doses of OPV and DTP vaccine at 6, 10 and 14 weeks of age. MV was scheduled at 9 months of age and booster doses of DTP and OPV were recommended at 18 months.

In addition to the routine vaccination program, 14 campaigns distributing MV, OPV and vitamin A supplementation (VAS) took place in Guinea-Bissau during the study period (Additional file 1: Table S1).

### Assessment of vaccination status and nutritional status

At all home visits, field assistants registered whether they had seen the vaccination card and if seen copied the vaccination dates. Children who possessed, but did not present a vaccination card could only enter at a later visit if the card was seen. Children were considered measles-unvaccinated if they presented a vaccination card without a MV registered or if they did not have a vaccination card and the mother stated that the child had never been vaccinated. Nutritional status was assessed by measurement of the mid-upper-arm circumference (MUAC). The mothers were asked about breastfeeding and supplementary feeding.

### Statistical analyses

We compared background information for measles-vaccinated children and measles-unvaccinated children included in the analysis using the Wilcoxon rank-sum test, χ^2^-test and t-test. The child MUAC is presented as z-score compared to the WHO reference [12].

We compared overall mortality between measles-vaccinated children and measles-unvaccinated children in Cox proportional hazards models with age as underlying time and stratified by cluster. Children entered the analysis on the date their vaccination card was inspected and remained under follow-up until the subsequent visit (when information on vital status was obtained) or for a maximum of 6 months. Thus, a child could contribute several, not overlapping observation periods with known vaccination status at the beginning of the period, while observation periods with unknown vaccination status at the beginning of the interval, were excluded. To asses at what age it was most beneficial to receive MV, measles-vaccinated children were divided in three groups according to their age at measles vaccination: Early (6–8 months (<=274 days)), recommended (9–11 months (275–365 days)) and late (> 11 months (> = 366 days)). Three percent of measles-vaccinated children (330/11,260) had a measles vaccination age before 6 months. These children were classified as vaccinated at 6 months in the analyses. Each child could contribute with several observations at different time points in the unvaccinated or the vaccinated groups according to the vaccination status at the beginning of the follow-up period. Since several studies have found sex-differential effects of MV [3, 9, 13–15], we stratified the analyses by sex. The results are presented as mortality rates (deaths per 1000 person years (PYRS)) and HRs with 95% confidence intervals. All background factors (Table 1) were included as covariates in the Cox model one by one to see if they altered the estimates by more than 10%. No estimate changed by more than 6% (Additional file 1: Table S2), and hence we base our conclusion on the unadjusted estimates. Among measles-vaccinated children, we modelled measles vaccination age as a continuous variable. Modelling the age at vaccination as a linear spline with four knots did not describe the data any better than the simple linear model (Likelihood Ratio test, *p* = 0.29) and we therefore describe mortality changes per month increase in measles vaccination age across the whole age span.

**Table 1:** Characteristics at the beginning of an observation period

	Measles unvaccinated	Measles Vaccinated	*P*-value
Number (%)	9440 (30)	22,285 (70)	
Age^a^ [median (IQR)]	10.9 (8.1–17.6)	22.6 (16.3–28.9)	<.0001
Sex [n (%)]			0.68
Male	4782 (51)	11,345 (51)	
Child MUAC (z-score)^b^ [mean (SD)]	−0.60 (1.15)	−0.66 (1.07)	<.0001
Region [n (%)]			<.0001
Oio	1930 (30)	4477 (70)	
Biombo	2287 (34)	4438 (66)	
Gabu	1685 (24)	5352 (76)	
Cacheu	1613 (31)	3524 (69)	
Bafata	1925 (30)	4494 (70)	
Ethnicity [n (%)]^c^			<.0001
Balanta	1701 (18)	3159 (14)	
Papel	1904 (20)	3587 (16)	
Mandinga, Fula	4419 (47)	12,268 (55)	
Mandjaco, mancanha	789 (8)	1615 (7)	
Others	578 (6)	1519 (7)	
Season of birth [n (%)]			.0008
Dry season	5215 (55)	12,770 (57)	
Maternal schooling [n (%)]^d^			0.08
0 years	6277 (66)	15,450 (69)	
1–4 years	1111 (12)	2522 (11)	
> 4 years	419 (4)	964 (4)	
Maternal age^e^ [mean(SD)]	26.1 (6.9)	25.9 (6.9)	0.01
Maternal MUAC^f^ [mean [SD]]	254 (25)	256 (1.07)	<.0001
Lives with mother^g^ [n (%)]			<.0001
Yes	9352 (99)	21,983 (99)	
No	73 (1)	293 (1)	
Stopped breastfeeding^h^ [n (%)]			<.0001
Yes	1116 (12)	8976 (40)	
No	8318 (88)	13,304 (60)	
Sleeping under a bed net^i^ [n (%)]			0.13
All year	3644 (39)	8882 (40)	
Rainy season only	5496 (58)	12,736 (57)	
No	280 (3)	645 (3)	
Year of card inspection [n (%)]			<.0001
1999	1002 (11)	2283 (10)	
2000	1202 (13)	2621 (12)	
2001	1528 (16)	2766 (12)	
2002	1524 (16)	2717 (12)	
2003	1165 (12)	3186 (14)	
2004	1113 (12)	3564 (16)	
2005	1373 (15)	3614 (16)	
2006	533 (6)	1535 (7)	

^a^Age in months at the date of card inspection

^b^Mean Z-score MUAC-for-age using the 2006 WHO reference (Standard deviation). Missing information for 550 observations from measles-unvaccinated and children and 988 observations from measles-vaccinated children

^c^Missing information for 49 observations from measles-unvaccinated children and 137 observations from measles-vaccinated children

^d^Missing information for 1633 observations from measles-unvaccinated children and 3351 observations from measles-vaccinated children

^e^Mean maternal age at the date of the child’s birth (Standard deviation). Missing information for 50 observations from measles-unvaccinated children and 130 observations from measles-vaccinated children

^f^Mean maternal MUAC at registration of pregnancy (Standard deviation). Missing information for 5576 observations from measles-unvaccinated children and 12,731 observations from measles-vaccinated children

^g^Missing information for 4 observations from measles-unvaccinated children and 3 observations from measles-vaccinated children

^h^Missing information for 5 observations from measles-unvaccinated children and 5 observations from measles-vaccinated children

^i^Missing information for 19 observations from measles-unvaccinated children and 22 observations from measles-vaccinated children

Abbreviations: *MUAC* mid-upper-arm-circumference

To disentangle the effect of early MV from the effect of campaigns with other vaccines or VAS, we conducted survival analyses in which follow-up time was split on the first day of the national campaigns. Since campaigns in Guinea-Bissau have had very high coverage [16], we did not rely on individual level information but split follow-up time at the time a child was eligible for a campaign. All children contribute observation time in the pre-campaign group until the date of the campaign and time in the after-campaign group after the split. We used Stata14 for the analyses.

## Results

### Study population

**Fig. 1:**
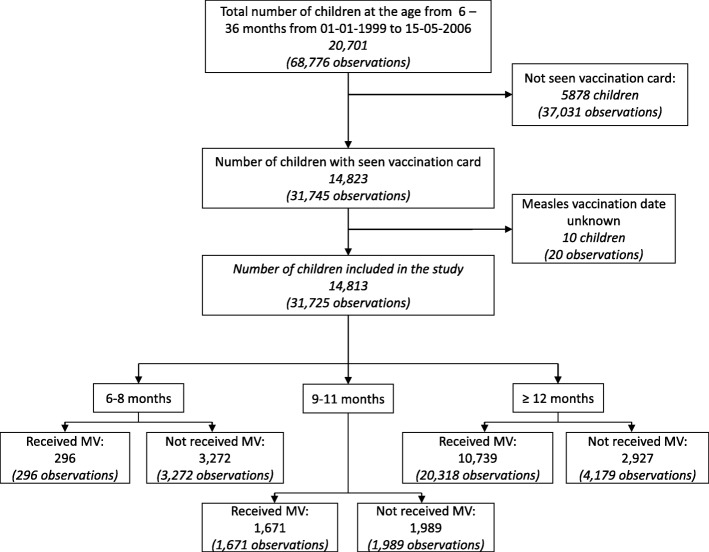
Flowchart illustrating study population

Between January 1st 1999 and May 15th 2006, BHP followed 20,701 children between 6 and 36 months of age in rural Guinea-Bissau of whom 14,823 (72%) had a vaccination card inspected. Excluding 10 children with unknown MV date, we retained 14,813 children contributing 31,725 observations in the analyses (Fig. 1). MV coverage increased with age, being 8% (296/3568) for children when they had their vaccination card seen between 6 and 8 months of age, 46% (1671/3660) for children when their card was inspected between 9 and 11 months and 77% (9898/12,825) when their card was first inspected between 12 and 36 months. Overall, children were measles vaccinated at 70% (22,285/31,725) of the observations (Fig. 1).

### Baseline characteristics

The observations from measles-unvaccinated children came from younger children (median age 10.9 months) than the observations from measles-vaccinated children (median age 22.6 months) (*p* < 0.0001). MUAC-for-age was lower for the measles-vaccinated than for the measles-unvaccinated children, but this difference disappeared when the comparison was stratified by age group (Additional file 1: Table S3). The proportion of measles-vaccinated children differed by region (p < 0.0001) and ethnicity (p < 0.0001). Furthermore, there were slight differences by season of birth (*p* = 0.0008), maternal age (*p* = 0.01) and maternal MUAC during pregnancy (p < 0.0001). Sex, sleeping under mosquito net and maternal schooling did not differ significantly between the two groups (Table 1, Additional file 1: Table S3).

### Mortality

Among all measles-vaccinated children, 23% (2560 children; 5740 observations) were vaccinated before 274 days of age, 43% (4846 children; 9706 observations) between 274 and 365 days of age and 34% (3854 children; 6839 observations) after 365 days of age (Table 2). The mortality rate was 45/1000 PYRS among measles-vaccinated children overall (Early-vaccinated: 42/1000 PYRS, recommended age: 44/1000 PYRS and late vaccinated: 48/1000 PYRS), whereas it was 66 among measles-unvaccinated children (Table 2). In spite of almost half of the measles-unvaccinated children receiving MV during follow-up (Table 2, Additional file 1), mortality among measles-vaccinated children was lower than among measles-unvaccinated children (HR 0.76 (95% CI: 0.63–0.91)). The difference in survival between measles-vaccinated and measles-unvaccinated children was statistically significant for early-vaccinated children (HR: 0.68 (0.53–0.87)) and children vaccinated at the recommended age (HR: 0.77 (0.62–0.96)), but not for late-vaccinated children (HR: 0.86 (0.67–1.11)) (Table 2). Censoring measles deaths (10%; 74/713) did not alter the conclusion (Additional file 1: Table S4).

**Table 2:** Mortality for children with and without measles vaccine and vaccines during follow-up. Stratified by age at measles vaccination and sex

	Number of observations	MR per 1000 PYRS (deaths/PYRS)	HR (95% CI)^a^	Number of observations with a seen vaccination card within 12 months after visit	Measles Vaccinated during follow up	DTP Vaccinated during follow up
All children						
Measles unvaccinated	9440	65.5 (272/4155)	1 (ref)	5825	48% (2767/5825)	30% (1721/5825)
Measles vaccinated	22,285	44.9 (441/9811)	0.76 (0.63–0.91)	16,535	3% (532/16535)	10% (1580/16535)
Early age (< 274 days)^b^	5740	41.9 (106/2528)	0.68 (0.53–0.87)	4096	4% (183/4096)	10% (424/4096)
Recommended age (274–365 days) ^b^	9706	44.3 (188/4245)	0.77 (0.62–0.96)	7027	3% (201/7027)	7% (489/7027)
Late (366–735 days) ^b^	6839	48.4 (147/3038)	0.86 (0.67–1.11)	5412	3% (148/5412)	12% (667/5412)
Boys						
Measles unvaccinated	4782	63.7 (134/2104)	1 (ref)	2943	47% (1385/2943)	28% (832/2943)
Measles vaccinated	11,345	46.2 (231/4995)	0.79 (0.62–1.00)	8505	3% (270/8505)	10% (814/8505)
Early age (< 274 days) ^b^	2936	48.1 (62/1290)	0.79 (0.57–1.08)	2074	4% (85/2074)	11% (228/2074)
Recommended age (274–365 days)^b^	4959	42.8 (93/2170)	0.75 (0.56–1.00)	3636	3% (107/3636)	7% (254/3636)
Late (366–735 days)^b^	3450	49.5 (76/1535)	0.89 (0.64–1.22)	2795	3% (78/2795)	12% (332/2795)
Girls						
Measles unvaccinated	4658	67.3 (138/2051)	1 (ref)	2882	48% (1382/2882)	31% (889/2882)
Measles vaccinated	10,940	43.6 (210/4816)	0.73 (0.57–0.93)	8030	3% (262/8030)	10% (766/8030)
Early age (< 274 days)^b^	2804	35.5 (44/1238)	0.57 (0.40–0.81)	2022	5% (98/2022)	10% (196/2022)
Recommended age (274–365 days)^b^	4747	45.8 (95/2075)	0.79 (0.59–1.05)	3391	3% (94/3391)	7% (235/3391)
Late (366–735 days)^b^	3389	47.2 (71/1503)	0.83 (0.60–1.15)	2617	3% (70/2617)	13% (335/2617)

^a^Age as underlying time scale, stratified by village cluster; ^b^Age at measles vaccination according to the information obtained from the vaccination card at the beginning of the observation period. Abbreviations: *MR* Mortality Rate, *PYRS* Person years, *HR* Hazard Ratio, *CI* Confidence interval

Limiting the analysis to the measles-vaccinated children and modelling the effect of MV-age linearly, mortality was 2.6% (0.4–5.1%) higher for each month the measles vaccination age increased.

Stratified by sex, measles-vaccinated children had lower mortality for both boys (HR = 0.79 (0.62–1.00)) and girls (HR = 0.73 (0.57–0.93), Table 2). The pattern of better survival with lower age at MV was evident for girls, the estimates being 0.57 (0.40–0.81) for early vaccination, 0.79 (0.59–1.05) for children vaccinated at 9–11 months and 0.83 (0.60–1.15) for children vaccinated after 12 months of age. The age of MV seemed of less importance in boys (Table 2), but we saw no evidence of an interaction between sex and age of MV when limiting the analysis to measles-vaccinated children (*p* = 0.93). Mortality tended to be lower for girls than boys among early-vaccinated children (Additional file 1).

### Campaign analyses

When observation time was split at the date of the first OPV campaign after inspecting the vaccination card, 31% of observation time was after OPV campaigns. Lower age at vaccination was associated with lower mortality before OPV campaigns, but less consistently so after OPV (Table 3). For the VAS campaigns, 27% of observation time was after campaigns. The pattern for the effect of age of MV was similar before and after VAS campaigns and the mortality did not differ significantly before and after the campaign (*p* = 0.34 for interaction between VAS campaign and vaccination status). The estimates were similar for boys and girls (Table 3, *p* = 0.86 for interaction between sex, VAS and MV status). Only 2–5% of children were exposed to the MV or meningitis campaigns and data were insufficient to assess the effect of age of MV after the meningitis and MV campaigns. Censoring at the MV and meningitis campaigns did not alter the conclusions (Additional file 1: Table S5).

**Table 3:** Mortality before and after OPV and VAS campaigns, Stratified by age at measles vaccination and sex

	OPV campaign				VAS campaign			
	Before		After		Before		After	
All children (number of observations)	31,725		13,936		31,725		11,950	
	MR per 1000 PYRS deaths/ PYRS)	HR (95% CI)^a^	MR per 1000 PYRS deaths/ PYRS)	HR (95% CI)^a^	MR per 1000 PYRS (deaths/ PYRS)	HR (95% CI)^a^	MR per 1000 PYRS (deaths/ PYRS)	HR (95% CI)^a^
Measles unvaccinated	73.2 (215/2938)	1 (ref)	46.8 (57/1217)	1 (ref)	72.2 (231/3199)	1 (ref)	42.9 (41/956)	1 (ref)
Measles vaccinated	47.2 (345/7314)	0.74 (0.60–0.92)	38.5 (96/2496)	0.85 (0.56–1.27)	47.3 (371/7840)	0.76 (0.61–0.93)	35.5 (70/1971)	0.71 (0.42–1.18)
Early age (< 274 days)^b^	45.7 (86/1883)	0.70 (0.53–0.92)	31.0 (20/645)	0.63 (0.36–1.12)	44.0 (90/2047)	0.68 (0.52–0.89)	33.3 (16/481)	0.69 (0.35–1.35)
Recommended age (274–365 days)^b^	43.2 (135/3124)	0.71 (0.55–0.92)	47.2 (53/1122)	1.07 (0.68–1.69)	45.5 (151/3319)	0.76 (0.60–0.97)	39.9 (37/926)	0.78 (0.45–1.36)
Late (366–735 days)^b^	53.7 (124/2308)	0.89 (0.67–1.18)	31.5 (23/730)	0.70 (0.39–1.25)	52.5 (130/2474)	0.88 (0.67–1.16)	30.1 (17/564)	0.57 (0.28–1.15)
Boys (number of observations)	16,127		7104		16,127		6107	
Measles unvaccinated	71.9 (107/1488)	1 (ref)	43.9 (27/616)	1 (ref)	70.5 (115/1632)	1 (ref)	40.3 (19/472)	1 (ref)
Measles vaccinated	47.5 (177/3725)	0.75 (0.57–0.99)	42.5 (54/1270)	0.97 (0.58–1.64)	49.6 (198/3993)	0.80 (0.61–1.04)	32.9 (33/1002)	0.73 (0.37–1.41)
Early age (< 274 days)^b^	49.1 (47/957)	0.76 (0.53–1.09)	45.1 (15/333)	0.93 (0.47–1.84)	49.8 (52/1045)	0.78 (0.55–1.10)	40.9 (10/245)	0.90 (0.39–2.09)
Recommended age (274–365 days)^b^	42.4 (68/1605)	0.69 (0.50–0.97)	44.2 (25/566)	1.08 (0.58–1.98)	45.8 (78/1702)	0.77 (0.56–1.05)	32.0 (15/469)	0.69 (0.32–1.51)
Late (366–735 days)^b^	53.3 (62/1164)	0.88 (0.61–1.26)	37.8 (14/371)	0.84 (0.41–1.75)	54.6 (68/1246)	0.92 (0.65–1.31)	27.7 (8/289)	0.57 (0.22–1.46)
Girls (number of observations)	15,598		6832		15,598		5843	
Measles unvaccinated	74.5 (108/1450)	1 (ref)	49.9 (30/601)	1 (ref)	74.0 (116/1567)	1 (ref)	45.4 (22/484)	1 (ref)
Measles vaccinated	46.8 (168/3589)	0.74 (0.56–0.97)	34.2 (42/1227)	0.72 (0.42–1.24)	45.0 (173/3847)	0.71 (0.54–0.93)	38.2 (37/969)	0.69 (0.36–1.31)
Early age (< 274 days)^b^	42.1 (39/926)	0.63 (0.43–0.93)	16.0 (5/312)	0.32 (0.12–0.86)	37.9 (38/1002)	0.58 (0.39–0.85)	25.4 (6/236)	0.48 (0.18–1.29)
Recommended age (274–365 days)^b^	44.1 (67/1519)	0.73 (0.52–1.02)	50.4 (28/556)	1.06 (0.59–1.90)	45.1 (73/1617)	0.75 (0.54–1.04)	48.1 (22/458)	0.83 (0.41–1.67)
Late (366–735 days)^b^	54.2 (62/1144)	0.90 (0.63–1.29)	25.1 (9/359)	0.54 (0.23–1.24)	50.5 (62/1228)	0.83 (0.59–1.19)	32.7 (9/275)	0.56 (0.22–1.40)

^a^Age as underlying time scale, stratified by village cluster; ^b^Age at measles vaccination according to the information obtained from the vaccination card at the beginning of the observation period

Abbreviations: *MR* Mortality Rate, *PYRS* Person years, *HR* Hazard Ratio, *CI* Confidence interval

## Discussion

### Main results

Measles-vaccinated children had a significantly lower mortality compared with measles-unvaccinated children. The benefit was stronger for early-vaccinated children.

### Strengths and weaknesses

The study was conducted within a well-established HDSS representative of the rural population of Guinea-Bissau. The data were collected from the five most populous regions in Guinea-Bissau, where a large cohort of over 14,000 children contributing more than 30,000 observations was followed. The data collection in the rural areas had been ongoing for 9 years at the initiation of the study, and the field workers were experienced.

We used the landmark approach, where children entered the analyses after vaccination status was assessed by inspecting a vaccination card. Hence, the measles-unvaccinated group was well defined. Furthermore, children could only change vaccination status from the date of seeing a vaccination card. This method eliminates the risk of survival bias but misclassifies some observation time for children vaccinated between two visits and therefore provides conservative estimates [17].

Receiving MV during follow-up could lead to bias in the estimated association between age of MV and mortality. Among the measles-vaccinated children, only 3% received a second dose of MV during follow-up and 10% received DTP, while more measles-unvaccinated children received MV (48%) and DTP (30%) during follow-up. However, the effect of age at vaccination remained evident when the analysis was limited to measles-vaccinated children, among whom few were vaccinated during follow-up.

The two groups differ with regard to many of the measured baseline characteristics. As with all observational studies, we cannot exclude confounding. However, adjusting for measured background factors did not alter the estimates by more than 6%. Furthermore, since the analyses were stratified by cluster and had age as underlying timescale, we only compared mortality of measles-vaccinated children with measles-unvaccinated children within the same village and of the same age. Therefore, the children compared are more similar than children from different villages, which may explain why adjusting for the background factors had little effect.

The cause of death classifications are based on maternal information. However, we presume that the data on deaths due to measles, a well-known and characteristic infection, are accurate. Frailty bias could occur in the study with sick children not being vaccinated and hence an increasingly frail group of unvaccinated children the longer after the recommended age of MV the vaccination cards were inspected. Reasons for not being vaccinated were not registered. However, if the differential survival was caused by frailty bias, the estimated effect of MV compared with unvaccinated should be weakened by adjusting for MUAC at the time of card inspection, which it did not (Supplementary Table 2). Furthermore, a differential survival should become stronger with age (as the unvaccinated children become an increasingly selected group). This was not the case; on the contrary, the differential survival was stronger in the younger children, indicating that it was not a frail subgroup, which did not receive MV.

### Consistency with previous studies

Several studies have indicated stronger beneficial effects of MV in girls than in boys [9, 13–15], as also concluded in the WHO review [3]. In our study, the effect may have been slightly stronger for girls than for boys. The WHO review did not conclude on the importance of age at vaccination, stating that the evidence was insufficient [3]. However, a number of studies have assessed the impact of age at vaccination. In line with our results, a review of data published until 2015 also revealed that early MV is more beneficial than later MV [10]. In a study from 1993, children receiving MV at 4–8 months had lower mortality than children vaccinated later. This could partly be explained by the early-vaccinated children being revaccinated. In our study, revaccination occurred in only 4% of early-vaccinated children and 3% of late-vaccinated children during follow-up. Hence, it is unlikely that the beneficial effects of early MV are explained by revaccination. There were few deaths after MV campaigns and we could therefore not evaluate whether the effect of early MV differed before and after the campaign.

Previous studies have indicated a beneficial effect of VAS campaigns with or after MV, but not of VAS with or after DTP. In an observational study of VAS campaigns, where vaccination status was documented, VAS was beneficial when MV was the most recent vaccine (HR for VAS vs. no-VAS = 0.34 (0.14–0.85)), while VAS was not associated with lower mortality when DTP was the most recent vaccine (HR = 1.29 (0.52–3.22)) [18]. In a campaign, where VAS was co-delivered with missing vaccines, children who had received VAS and DTP had higher mortality than children who had received VAS and MV simultaneously (p for homogeneity = 0.0005) [19]. In our study, we did not find any difference in the effect of age at MV before and after the VAS campaign. Nor did we see a sex-differential effect of VAS campaigns. In a randomized controlled trial of VAS at vaccination contacts, where 63% received VAS or placebo with MV, we found that VAS was beneficial for girls (HR = 0.45 (0.24–0.87), but not for boys (HR = 1.92 (0.98–3.75) [20]. Whether the contrasting effects should be explained by timing of the interventions relative to each other, or possibly co-delivered interventions (i.e. OPV) should be studied in more detail.

### Interpretation and implications

Our study suggests that early MV lowers mortality, but giving early MV is only recommended under special circumstances [21] to avoid interference from MatAb on the antibody response to MV [4]. In a randomized trial, a two-dose vaccination strategy with MV at 4½ and 9 months vs. one dose at 9 months resulted in lower antibody titer at 24 months of age, but 97% still obtained protective antibody levels [22]. However, though the antibody response may be blunted, MV given in presence of MatAb still prevents measles, especially severe measles and measles mortality [23]. Hence, the sole focus on measles antibodies as marker of the effect of MV may obstruct the best use of the vaccine. The policy should be based on the impact of early MV on child health: If early MV in the presence of MatAb lowers mortality, as the present data and a previous study from Guinea-Bissau indicate [8], and does not impair protection against measles [23], early MV should be policy.

The current policy is that the age of MV should increase when measles infection is under control. Our study suggests that this may lead to increased overall child mortality. Children will not benefit from the beneficial NSEs at a younger age and will not benefit from the additional NSEs of early MV. In contrast, it might decrease mortality further, if an early first dose followed by a second booster dose around 9 months of age was introduced. However, further assessment of the effect of early MV is needed.

## Conclusion

Measles-vaccinated children had a significantly better survival compared with measles-unvaccinated children, the beneficial effects being strongest for early-vaccinated children. Given the beneficial effects of MV, the current policy to increase the age of vaccination when measles control improves may not optimize the impact of MV on mortality. The beneficial NSEs of MV and the differential effect of MV by age at vaccination should be considered when planning vaccination programs in low-income countries.

## Additional file

Additional file 1: Supplementary Results. **Table S1.** List of vaccination campaigns in Guinea-Bissau 1999–2006. **Table S2.** Mortality of children with and without measles vaccine with information on potential confounders. Stratified by age of vaccination. **Table S3.** Characteristics at the beginning of an observation period for children by age at entry into the analyses. **Table S4.** Mortality of children with and without measles vaccine with deaths due to measles censored. Stratified by age of vaccination and by sex. **Table S5.** Mortality before meningitis and measles vaccinations campaigns. Stratified by age at measles vaccination and by sex. **Table S6.** Mortality of measles-vaccinated children with MV as most recent vaccine, and measles-unvaccinated children with DTP as most recent vaccine. Stratified by age at measles vaccination and by sex. **Table S7.** Mortality of children with and without measles vaccine. Children with no vaccines at all are excluded. Stratified by age at measles vaccination and by sex. (DOCX 45 kb)

## Abbreviations

BCG: Bacillus Calmette-Guérin vaccine
BHP: Bandim Health Project
CI: Confidence interval
DTP: Diphtheria-tetanus-pertussis vaccine
HDSS: Health and Demographic Surveillance System
HR: Hazard ratio
MatAb: Maternal measles antibodies
MUAC: Mid-upper-arm circumference
MV: Measles vaccine
NSE: Non-specific effects
OPV: Oral polio vaccine
PYRS: Person years
VAS: Vitamin A supplementation
