# Supplementary material for: Is early measles vaccination associated with stronger survival benefits than later measles vaccination?
Source: BMC Public Health. 2018 Aug 7;18:984. doi: 10.1186/s12889-018-5866-y (PMC6081866; doi:10.1186/s12889-018-5866-y)
Supplement: Supplementary file 1 — Supplementary Results. Table S1. List of vaccination campaigns in Guinea-Bissau 1999–2006. Table S2. Mortality of children with and without measles vaccine with information on potential confounders. Stratified by age of vaccination. Table S3. Characteristics at the beginning of an observation period for children by age at entry into the analyses. Table S4. Mortality of children with and without measles vaccine with deaths due to measles censored. Stratified by age of vaccination and by sex. Table S5. Mortality before meningitis and measles vaccinations campaigns. Stratified by age at measles vaccination and by sex. Table S6. Mortality of measles-vaccinated children with MV as most recent vaccine, and measles-unvaccinated children with DTP as most recent vaccine. Stratified by age at measles vaccination and by sex. Table S7. Mortality of children with and without measles vaccine. Children with no vaccines at all are excluded. Stratified by age at measles vaccination and by sex. (DOCX 45 kb) [file 12889_2018_5866_MOESM1_ESM.docx]

**Supplementary Material**

**Is early measles vaccination associated with stronger survival benefits than later measles vaccination?**

**Supplementary results**

**Censoring deaths due to measles**

Censoring deaths classified as due to measles infection (40 unvaccinated children, 34 measles vaccinated) did not alter the conclusions. The overall HR comparing measles-vaccinated with measles-unvaccinated children was 0.79 (0.65-0.95)) and the association was still particularly strong for children vaccinated before 9 months of age (Supplementary Table 4).

**Vaccinations during follow-up**

We classified children according to their vaccination status at the beginning of follow-up, and kept vaccination status fixed through the 6 months of follow-up. Vaccines administered during follow-up could have modified the estimated differences between measles-vaccinated and measles-unvaccinated children and we therefore examined how many children received other vaccines during follow-up (Table 2). Among children with an inspected vaccination card within 12 months of the beginning of follow-up, 48% (2,767/5,825) of the initially measles-unvaccinated children received MV during follow-up, whereas 30% (1,721/5,825) received DTP during follow-up. Among the measles-vaccinated children, 10% (1580/16,535) received DTP during follow-up, whereas only 3% (532/16,535) received a second dose of routine MV during follow-up (Table 2). Stratifying by sex revealed that the same proportion of measles-unvaccinated boys and girls received MV during follow-up, while 28% (832/2,943) of the boys and 31% (889/2,882) of the girls received DTP during follow-up, p=0.03 (Table 2).

**Sequence of vaccinations**

Limiting the analysis to the 57% (8,440 children, 16,726 observations) who had received the vaccines in the recommended sequence, i.e. measles-vaccinated children having MV as the most recent vaccine and measles-unvaccinated children having DTP as the most recent vaccine, revealed a similar pattern as the main analysis; effect of age at vaccination being evident mainly for girls. However, the differential survival was less pronounced (Supplementary Table 6). Excluding 800 observations for children not having received any vaccines revealed the same pattern (Supplementary Table 7).

**Female/Male mortality**

Previous studies have suggested that MV may have a particularly beneficial effect for girls. We therefore examined the female/male (F/M) HR among different age and vaccination groups. Among measles-unvaccinated children, the F/M HR was 1.02 (0.80-1.29). For children who received MV early the F/M HR was 0.74 (0.50-1.09), whereas the F/M HR was 1.07 (0.80-1.43) for children vaccinated at 9-11 months and 0.95 (0.69-1.32) for children vaccinated after 12 months of age.

**Limiting the analysis to children <12 months**

We have previously reported that MV was associated with lower mortality among children aged >12 months in 1999-2006[17], thus in a cohort which includes the oldest children in the present cohort. Among children less than 12 months of age at the beginning of follow up, MV was associated with a HR of 0.83 (0.59-1.17); 0.92 (0.60-1.43) for boys and 0.72 (0.43-1.20) for girls.

**Supplementary table 1: List of vaccination campaigns in Guinea-Bissau 1999-2006**

| **Year** | **Month** | **Type of campaign** |
| --- | --- | --- |
| 1999 | March | Meningitis (national – all ages) |
| 1999 | October | OPV (0-59 months) and VAS (6-59 months) |
| 1999 | December | Measles (national) and OPV (0-59 months) |
| 2000 | October | OPV (0-59 months) |
| 2001 | October | OPV (0-59 months) |
| 2001 | November | OPV (0-59 months) and VAS (6-59 months) |
| 2002 | October | OPV (0-59 months) |
| 2002 | November | OPV (0-59 months) and VAS (6-59 months) |
| 2003 | November | VAS (6-59 months) and missing routine vaccines |
| 2004 | October | OPV (0-59 months) |
| 2004 | November | OPV (0-59 months) and VAS (6-59 months) |
| 2005 | November | OPV (0-59 months) |
| 2005 | December | OPV (0-59 months) and VAS (6-59 months) |
| 2006 | May | VAS (6-59 months), Mebendazol (12-59 months)  and MV (6 months – 15 years) |

Abbreviations: OPV: Oral Polio vaccine; VAS: Vitamin A Supplementation; MV: Measles Vaccine

**Supplementary Table 2: Mortality of children with and without measles vaccine with information on potential confounders. Stratified by age of vaccination.**

|  | Number of observations with information | MR per 1000 PYRS (deaths/PYRS) | HR (95% CI)^1^ | HR adjusted for potential confounder (95% CI)^1^ |
| --- | --- | --- | --- | --- |
| **Sex** |  |  |  |  |
| Measles unvaccinated | 9440 | 65.5 (272 / 4155) | 1 (ref) | 1 (ref) |
| Measles vaccinated | 22285 | 45.0 (441 / 9809) | 0.76 (0.63-0.91) | 0.76 (0.63-0.91) |
| Early age (<274 days*)* | 5740 | 41.9 (106 / 2527) | 0.68 (0.53-0.87) | 0.68 (0.53-0.87) |
| Recommended age (274-365 days) | 9706 | 44.3 (188 / 4244) | 0.77 (0.62-0.96) | 0.77 (0.62-0.96) |
| Late (366-735 days) | 6839 | 48.4 (147 / 3038) | 0.86 (0.67-1.11) | 0.86 (0.67-1.11) |
| **MUAC for age^2^** |  |  |  |  |
| Measles unvaccinated | 8890 | 63.4 (248 / 3913) | 1 (ref) | 1 (ref) |
| Measles vaccinated | 21297 | 43.1 (404 / 9374) | 0.76 (0.63-0.93) | 0.78 (0.64-0.95) |
| Early age (<274 days*)* | 5456 | 40.8 (98 / 2402) | 0.69 (0.54-0.90) | 0.71 (0.55-0.92) |
| Recommended age (274-365 days) | 9279 | 42.6 (173 / 4056) | 0.78 (0.62-0.97) | 0.80 (0.63-1.00) |
| Late (366-735 days) | 6562 | 45.6 (133 / 2916) | 0.85 (0.66-1.11) | 0.84 (0.65-1.09) |
| **Ethnicity^3^** |  |  |  |  |
| Measles unvaccinated | 9391 | 64.8 (268 / 4135) | 1 (ref) | 1 (ref) |
| Measles vaccinated | 22148 | 45.1 (440 / 9753) | 0.77 (0.63-0.93) | 0.77 (0.64-0.93) |
| Early age (<274 days*)* | 5712 | 42.1 (106 / 2515) | 0.69 (0.54-0.88) | 0.69 (0.54-0.88) |
| Recommended age (274-365 days) | 9645 | 44.6 (188 / 4220) | 0.78 (0.63-0.97) | 0.78 (0.63-0.97) |
| Late (366-735 days) | 6791 | 48.4 (146 / 3017) | 0.86 (0.67-1.11) | 0.87 (0.67-1.11) |
| **Season of birth^4^** |  |  |  |  |
| Measles unvaccinated | 9440 | 65.5 (272 / 4155) | 1 (ref) | 1 (ref) |
| Measles vaccinated | 22285 | 45 (441 / 9809) | 0.76 (0.63-0.91) | 0.76 (0.63-0.92) |
| Early age (<274 days*)* | 5740 | 41.9 (106 / 2527) | 0.68 (0.53-0.87) | 0.69 (0.54-0.88) |
| Recommended age (274-365 days) | 9706 | 44.3 (188 / 4244) | 0.77 (0.62-0.96) | 0.77 (0.62-0.96) |
| Late (366-735 days) | 6839 | 48.4 (147 / 3038) | 0.86 (0.67-1.11) | 0.86 (0.67-1.10) |
| **Maternal education^5^** |  |  |  |  |
| Measles unvaccinated | 7807 | 62.4 (215 / 3448) | 1 (ref) | 1 (ref) |
| Measles vaccinated | 18936 | 43 (359 / 8342) | 0.79 (0.64-0.97) | 0.79 (0.64-0.97) |
| Early age (<274 days*)* | 4788 | 38 (80 / 2107) | 0.67 (0.51-0.88) | 0.67 (0.51-0.88) |
| Recommended age (274-365 days) | 8194 | 45.2 (162 / 3585) | 0.85 (0.67-1.09) | 0.86 (0.67-1.09) |
| Late (366-735 days) | 5954 | 44.2 (117 / 2650) | 0.84 (0.63-1.11) | 0.84 (0.63-1.11) |
| **Maternal Age^6^** |  |  |  |  |
| Measles unvaccinated | 9390 | 64.8 (268 / 4134) | 1 (ref) | 1 (ref) |
| Measles vaccinated | 22155 | 45.1 (440 / 9755) | 0.77 (0.63-0.93) | 0.76 (0.63-0.92) |
| Early age (<274 days*)* | 5715 | 42.1 (106 / 2517) | 0.69 (0.54-0.88) | 0.68 (0.53-0.87) |
| Recommended age (274-365 days) | 9644 | 44.6 (188 / 4219) | 0.78 (0.63-0.97) | 0.77 (0.62-0.96) |
| Late (366-735 days) | 6796 | 48.3 (146 / 3020) | 0.86 (0.67-1.11) | 0.86 (0.67-1.11) |
| **Maternal MUAC at registration of pregnancy^7^** |  |  |  |  |
| Measles unvaccinated | 3864 | 60 (103 / 1716) | 1 (ref) | 1 (ref) |
| Measles vaccinated | 9557 | 49.4 (209 / 4234) | 0.96 (0.71-1.29) | 0.96 (0.72-1.29) |
| Early age (<274 days*)* | 2551 | 45.2 (51 / 1127) | 0.84 (0.58-1.21) | 0.85 (0.58-1.22) |
| Recommended age (274-365 days) | 4125 | 53.2 (97 / 1825) | 1.06 (0.76-1.48) | 1.06 (0.76-1.48) |
| Late (366-735 days) | 2881 | 47.6 (61 / 1282) | 0.97 (0.65-1.44) | 0.97 (0.65-1.44) |
| **Living with the mother** |  |  |  |  |
| Measles unvaccinated | 9425 | 65.3 (271 / 4150) | 1 (ref) | 1 (ref) |
| Measles vaccinated | 22276 | 44.9 (440 / 9807) | 0.76 (0.63-0.91) | 0.76 (0.63-0.91) |
| Early age (<274 days*)* | 5739 | 41.9 (106 / 2527) | 0.68 (0.53-0.87) | 0.68 (0.53-0.87) |
| Recommended age (274-365 days) | 9700 | 44.1 (187 / 4242) | 0.77 (0.62-0.95) | 0.77 (0.62-0.95) |
| Late (366-735 days) | 6837 | 48.4 (147 / 3037) | 0.86 (0.67-1.10) | 0.86 (0.67-1.10) |
| **Stopped breastfeeding** |  |  |  |  |
| Measles unvaccinated | 9434 | 65.5 (272 / 4153) | 1 (ref) | 1 (ref) |
| Measles vaccinated | 22280 | 44.9 (440 / 9808) | 0.76 (0.63-0.91) | 0.75 (0.62-0.91) |
| Early age (<274 days*)* | 5738 | 42 (106 / 2526) | 0.68 (0.53-0.87) | 0.68 (0.53-0.86) |
| Recommended age (274-365 days) | 9705 | 44.3 (188 / 4244) | 0.77 (0.62-0.96) | 0.77 (0.62-0.95) |
| Late (366-735 days) | 6837 | 48.1 (146 / 3037) | 0.86 (0.67-1.10) | 0.85 (0.66-1.10) |
| **Sleeping under bed net^8^** |  |  |  |  |
| Measles unvaccinated | 9420 | 65.6 (272 / 4148) | 1 (ref) | 1 (ref) |
| Measles vaccinated | 22263 | 44.8 (439 / 9803) | 0.75 (0.62-0.91) | 0.75 (0.62-0.91) |
| Early age (<274 days*)* | 5736 | 42 (106 / 2526) | 0.68 (0.53-0.87) | 0.68 (0.53-0.87) |
| Recommended age (274-365 days) | 9693 | 44.1 (187 / 4241) | 0.77 (0.62-0.95) | 0.77 (0.62-0.95) |
| Late (366-735 days) | 6834 | 48.1 (146 / 3036) | 0.85 (0.67-1.10) | 0.85 (0.66-1.10) |
| **Year of card inspection^9^** |  |  |  |  |
| Measles unvaccinated | 9440 | 65.5 (272 / 4155) | 1 (ref) | 1 (ref) |
| Measles vaccinated | 22285 | 45 (441 / 9809) | 0.76 (0.63-0.91) | 0.78 (0.65-0.94) |
| Early age (<274 days*)* | 5740 | 41.9 (106 / 2527) | 0.68 (0.53-0.87) | 0.70 (0.55-0.90) |
| Recommended age (274-365 days) | 9706 | 44.3 (188 / 4244) | 0.77 (0.62-0.96) | 0.81 (0.65-1.01) |
| Late (366-735 days) | 6839 | 48.4 (147 / 3038) | 0.86 (0.67-1.11) | 0.85 (0.66-1.09) |

1: Age as underlying time scale, stratified by village cluster

2: Z-score MUAC-for-age using the 2006 WHO reference, continouos.

3: Modelled as a categorical variable: Balanta, Papel, Mandinga/Fula, Mandjaco/Mancanha or Other

4: Season of birth, categorical: Rainy: June-November; Dry: December-May

5: Modelled as a categorical variable: None, 1-4 years; >4 years

6: Maternal age at date of child’s birth, continuous

7: Maternal MUAC at registration of pregnancy. Assessed only for mothers seen during pregnancy. Continuous.

8: Modelled as a categorical variable: No, Rainy season only, whole year.

9: Modelled as a categorical variable with each year defining a group.

Abbreviations: MR: Mortality Rate; PYRS: Person years; HR: Hazard Ratio; CI: Confidence interval; MUAC: mid-upper-arm-circumference

**Supplementary Table 3: Characteristics at the beginning of an observation period for children by age at entry into the analyses**

|  | **6-8 months** | | ***P-value*** | **9-11 months** | | ***P-value*** | **12 -36 months** | | ***P-value*** |
| --- | --- | --- | --- | --- | --- | --- | --- | --- | --- |
|  | **Measles-unvaccinated** | **Measles-vaccinated** |  | **Measles-unvaccinated** | **Measles-vaccinated** |  | **Measles-unvaccinated** | **Measles-vaccinated** |  |
| **Number (%)** | 3272 (92) | 296 (8) |  | 1989 (54) | 1671 (46) |  | 4179 (17) | 20318 (83) |  |
| **Age^1^** | 7.5 (6.7-8.2) | 8.1 (7.3-8.5) | <.0001 | 10.2 (9.5-11.0) | 10.7 (10.1-11.4) | <.0001 | 18.9 (14.7-25.1) | 23.7 (18.0-29.4) | <.0001 |
| **Sex [n (%)]** |  |  | 0.08 |  |  | 0.54 |  |  | 0.67 |
| Male | 1635 (50) | 164 (55) |  | 1039 (52) | 856 (51) |  | 2108 (50) | 10325 (51) |  |
| **Child Z-score MUAC-for-age ^2^** | -0.44 (1.19) | -0.47 (1.10) | 0.74 | -0.64 (1.15) | -0.57 (1.15) | 0.07 | -0.70 (1.10) | -0.67 (1.07) | 0.07 |
| **Region [n (%)]** |  |  | <.0001 |  |  | <.0001 |  |  | <.0001 |
| Oio | 684 (21) | 30 (10) |  | 398 (20) | 311 (19) |  | 848 (20) | 4136 (20) |  |
| Biombo | 770 (24) | 93 (32) |  | 406 (20) | 409 (24) |  | 1111 (27) | 3936 (19) |  |
| Gabu | 540 (17) | 121 (41) |  | 413 (21) | 430 (26) |  | 732 (18) | 4801 (24) |  |
| Cacheu | 573 (18) | 28 (9) |  | 326 (16) | 267 (16) |  | 714 (17) | 3229 (16) |  |
| Bafata | 705 (22) | 24 (8) |  | 446 (22) | 254 (15) |  | 774 (19) | 4216 (21) |  |
| **Ethnicity [n (%)]** |  |  | 0.0005 |  |  | .0009 |  |  | <.0001 |
| Balanta | 507 (15) | 43 (14) |  | 337 (17) | 221 (13) |  | 857 (21) | 2895 (14) |  |
| Papel | 648 (20) | 73 (25) |  | 321 (16) | 336 (20) |  | 935 (22) | 3178 (16) |  |
| Mandinga/Fula | 1576 (48) | 158 (53) |  | 1049 (53) | 852 (51) |  | 1794 (43) | 11258 (55) |  |
| Mandjaco/mancanha | 314 (10) | 10 (3) |  | 155 (8) | 152 (9) |  | 320 (8) | 1453 (7) |  |
| Others | 214 (7) | 11 (4) |  | 118 (6) | 106 (6) |  | 246 (6) | 1402 (7) |  |
| Season of birth [n (%)] |  |  | 0.05 |  |  | 0.75 |  |  | 0.91 |
| **Dry season** | 1678 (51) | 135 (45) |  | 1128 (57) | 939 (56) |  | 2409 (58) | 11696 (58) |  |
| **Maternal schooling [n (%)]** |  |  | 0.24 |  |  | .006 |  |  | 0.16 |
| 0 years | 2099 (64) | 194 (65) |  | 1338 (67) | 1063 (64) |  | 2840 (68) | 14193 (70) |  |
| 1-4 years | 420 (13) | 28 (9) |  | 211 (11) | 231 (14) |  | 480 (11) | 2263 (11) |  |
| > 4 years | 176 (5) | 13 (4) |  | 91 (5) | 86 (5) |  | 152 (4) | 865 (4) |  |
| **Maternal age^3^** | 26.0 (6.9) | 25.4 (6.3) | 0.11 | 26.0 (6.9) | 25.6 (6.7) | 0.10 | 26.3 (6.9) | 25.9 (6.9) | 0.003 |
| **Maternal MUAC^4^** | 255 (25) | 253 (26) | 0.26 | 253 (25) | 253 (25) | 0.96 | 253 (25) | 252 (25) | 0.024 |
| **Living with mother [n (%)]** |  |  | 0.30 |  |  | 0.91 |  |  | 0.43 |
| Yes | 3257 (100) | 295 (100) |  | 1976 (99) | 1661 (100) |  | 4119 (99) | 20027 (99) |  |
| No | 12 (0) | 0 (0) |  | 9 (0) | 8 (0) |  | 52 (1) | 285 (1) |  |
| **Stopped breastfeeding [n (%)]** |  |  | 0.48 |  |  | 0.88 |  |  | <.0001 |
| Yes | 12 (0) | 1 (1) |  | 9 (1) | 8 (1) |  | 1093 (26) | 8965 (44) |  |
| No | 3257 (100) | 295 (100) |  | 1976 (99) | 1661 (100) |  | 3082 (74) | 11350 (56) |  |
| **Sleeping under bed net [n (%)]** |  |  | 0.004 |  |  | 0.41 |  |  | 0.02 |
| All year | 1236 (38) | 83 (28) |  | 754 (38) | 615 (37) |  | 1654 (40) | 8184 (40) |  |
| Rainy season | 1952 (60) | 204 (69) |  | 1178 (59) | 998 (60) |  | 2366 (57) | 11534 (57) |  |
| No | 75 (2) | 8 (3) |  | 53 (3) | 56 (3) |  | 152 (4) | 581 (3) |  |
| **Year of card inspection [n (%)]** |  |  | <.0001 |  |  | <.0001 |  |  | <.0001 |
| 1999 | 254 (8) | 33 (11) |  | 206 (10) | 136 (8) |  | 542 (13) | 2114 (10) |  |
| 2000 | 403 (12) | 61 (21) |  | 255 (13) | 180 (11) |  | 544 (13) | 2380 (12) |  |
| 2001 | 458 (14) | 29 (10) |  | 358 (18) | 175 (10) |  | 712 (17) | 2562 (13) |  |
| 2002 | 441 (13) | 26 (9) |  | 271 (14) | 184 (11) |  | 812 (19) | 2507 (12) |  |
| 2003 | 434 (13) | 53 (18) |  | 264 (13) | 245 (15) |  | 467 (11) | 2888 (14) |  |
| 2004 | 502 (15) | 61 (21) |  | 245 (12) | 328 (20) |  | 366 (9) | 3175 (16) |  |
| 2005 | 597 (18) | 23 (8) |  | 282 (14) | 274 (16) |  | 494 (12) | 3317 (16) |  |
| 2006 | 183 (6) | 10 (3) |  | 108 (5) | 149 (9) |  | 242 (6) | 1375 (7) |  |

^1^ Median (interquartile range)

^2^ Mean Z-score MUAC for age using the 2006 WHO reference (standard deviation). Missing information for 550 observations from measles-unvaccinated and children and 988 observations from measles-vaccinated children

^3^ Mean maternal age at date of child’s birth (standard deviation). Missing information for 50 observations from measles-unvaccinated children and 130 observations from measles-vaccinated children

^4^ Mean maternal MUAC at registration of pregnancy (standard deviation). Missing information for 5576 observations from measles-unvaccinated children and 12,731 observations from measles-vaccinated children

Abbreviations: MUAC: mid-upper-arm-circumference

**Supplementary Table 4: Mortality of children with and without measles vaccine with deaths due to measles censored. Stratified by age of vaccination and by sex.**

|  | **Number of observations** | **MR per 1000 PYRS (deaths/PYRS)** | **HR (95% CI)^1^** |
| --- | --- | --- | --- |
| **All children** |  |  |  |
| Measles unvaccinated | 9440 | 61.6 (256/4155) | 1 (ref) |
| Measles vaccinated | 22285 | 44.1 (433/9811) | 0.79 (0.65-0.95) |
| Early age (<274 days) | 5740 | 40.8 (103/2528) | 0.70 (0.55-0.90) |
| Recommended age (274-365 days) | 9706 | 43.6 (185/4245) | 0.80 (0.64-1.01) |
| Late (366-735 days) | 6839 | 47.7 (145/3038) | 0.90 (0.70-1.16) |
| **Boys** |  |  |  |
| Measles unvaccinated | 4782 | 59.9 (126/2104) | 1 (ref) |
| Measles vaccinated | 11345 | 45 (225/4995) | 0.81 (0.64-1.04) |
| Early age (<274 days) | 2936 | 45.7 (59/1290) | 0.80 (0.58-1.10) |
| Recommended age (274-365 days) | 4959 | 41.9 (91/2170) | 0.78 (0.58-1.05) |
| Late (366-735 days) | 3450 | 48.9 (75/1535) | 0.93 (0.67-1.29) |
| **Girls** |  |  |  |
| Measles unvaccinated | 4658 | 63.4 (130/2051) | 1 (ref) |
| Measles vaccinated | 10940 | 43.2 (208/4816) | 0.76 (0.59-0.98) |
| Early age (<274 days) | 2804 | 35.5 (44/1238) | 0.60 (0.42-0.86) |
| Recommended age (274-365 days) | 4747 | 45.3 (94/2075) | 0.83 (0.62-1.11) |
| Late (366-735 days) | 3389 | 46.6 (70/1503) | 0.87 (0.62-1.20) |

1: Age as underlying time scale, stratified by village cluster

Abbreviations: MR: Mortality Rate; PYRS: Person years; HR: Hazard Ratio; CI: Confidence interval

**Supplementary Table 5: Mortality before meningitis and measles vaccinations campaigns. Stratified by age at measles vaccination and by sex.**

|  | **Hazard Ratios (95 % CI)^1^** | |
| --- | --- | --- |
|  | **Meningitis campaign** | **MV campaign** |
| **All Children (number of observations)** | 31,725 | 31,725 |
| Measles unvaccinated | 1 (ref) | 1 (ref) |
| Measles vaccinated | 0.74 (0.61-0.89) | 0.77 (0.63-0.93) |
| Early age (<274 days*)* | 0.67 (0.52-0.85) | 0.68 (0.53-0.88) |
| Recommended age (274-365 days) | 0.75 (0.60-0.93) | 0.78 (0.63-0.98) |
| Late (366-735 days) | 0.83 (0.65-1.07) | 0.86 (0.67-1.11) |
| **Boys (number of observations)** | 16,127 | 16,127 |
| Measles unvaccinated | 1 (ref) | 1 (ref) |
| Measles vaccinated | 0.76 (0.60-0.97) | 0.80 (0.63-1.03) |
| Early age (<274 days) | 0.77 (0.56-1.07) | 0.80 (0.58-1.10) |
| Recommended age (274-365 days) | 0.72 (0.54-0.97) | 0.77 (0.58-1.04) |
| Late (366-735 days) | 0.85 (0.62-1.18) | 0.91 (0.65-1.25) |
| **Girls (number of observations)** | 15,598 | 15,598 |
| Measles unvaccinated | 1 (ref) | 1 (ref) |
| Measles vaccinated | 0.71 (0.56-0.91) | 0.73 (0.57-0.93) |
| Early age (<274 days) | 0.56 (0.39-0.80) | 0.57 (0.40-0.81) |
| Recommended age (274-365 days) | 0.77 (0.58-1.03) | 0.79 (0.59-1.06) |
| Late (366-735 days) | 0.81 (0.59-1.13) | 0.82 (0.59-1.14) |

1: Age as underlying time scale, stratified by village cluster

Abbreviations: MR: Mortality Rate; PYRS: Person years; HR: Hazard Ratio; CI: Confidence interval

**Supplementary Table 6: Mortality of measles-vaccinated children with MV as most recent vaccine, and measles-unvaccinated children with DTP as most recent vaccine. Stratified by age at measles vaccination and by sex.**

|  | **Number of observations** | **MR per 1000 PYRS (deaths/PYRS)** | **HR (95% CI)^1^** | **Number of observations with a seen vaccination card within 12 months after visit** | **Measles Vaccinated during follow up in %** | **DTP Vaccinated during follow up** |
| --- | --- | --- | --- | --- | --- | --- |
| **All children** |  |  |  |  |  |  |
| Measles unvaccinated | 5579 | 59.2 (145/2450) | 1 (ref) | 3433 | 32% (1092/3433) | 23% (803/3433) |
| Measles vaccinated | 11147 | 43.0 (209/4863) | 0.86 (0.65-1.14) | 6351 | 2% (134/6351) | 1% (66/6351) |
| Early age (<274 days) | 2746 | 40.8 (49/1202) | 0.78 (0.55-1.12) | 1539 | 3% (42/1539) | 1% (18/1539) |
| Recommended age (274-365 days) | 5608 | 41.6 (101/2429) | 0.86 (0.63-1.17) | 3490 | 2% (66/3490) | 1% (32/3490) |
| Late (366-735 days) | 2793 | 47.9 (59/1233) | 1.04 (0.71-1.53) | 1322 | 2% (26/1322) | 1% (16/1322) |
| **Boys** |  |  |  |  |  |  |
| Measles unvaccinated | 2836 | 64.3 (80/1245) | 1 (ref) | 1741 | 32% (557/1741) | 21% (374/1741) |
| Measles vaccinated | 5738 | 46.0 (115/2500) | 0.83 (0.59-1.16) | 3287 | 2% (73/3287) | 1% (30/3287) |
| Early age (<274 days) | 1437 | 47.9 (30/626) | 0.82 (0.52-1.29) | 789 | 3% (21/789) | 1% (7/789) |
| Recommended age (274-365 days) | 2867 | 42.7 (53/1242) | 0.78 (0.53-1.16) | 1822 | 2% (38/1822) | 1% (17/1822) |
| Late (366-735 days) | 1434 | 50.6 (32/633) | 1.02 (0.64-1.65) | 676 | 2% (14/676) | 1% (6/676) |
| **Girls** |  |  |  |  |  |  |
| Measles unvaccinated | 2743 | 53.9 (65/1205) | 1 (ref) | 1692 | 32% (535/1692) | 25% (429/1692) |
| Measles vaccinated | 5409 | 39.8 (94/2363) | 0.90 (0.62-1.30) | 3064 | 2% (61/3064) | 1% (36/3064) |
| Early age (<274 days) | 1309 | 33.0 (19/576) | 0.72 (0.42-1.24) | 750 | 3% (21/750) | 1% (11/750) |
| Recommended age (274-365 days) | 2741 | 40.4 (48/1187) | 0.95 (0.62-1.44) | 1668 | 2% (28/1668) | 1% (15/1668) |
| Late (366-735 days) | 1359 | 45.0 (27/600) | 1.06 (0.63-1.78) | 646 | 2% (12/646) | 2% (10/646) |

1: Age as underlying time scale, stratified by village cluster

Abbreviations: MR: Mortality Rate; PYRS: Person years; HR: Hazard Ratio; CI: Confidence interval

**Supplementary Table 7: Mortality of children with and without measles vaccine. Children with no vaccines at all are excluded. Stratified by age at measles vaccination and by sex.**

|  | **Number of observations** | **MR per 1000 PYRS (deaths/PYRS)** | **HR (95% CI)^1^** |
| --- | --- | --- | --- |
| **All children** |  |  |  |
| Measles unvaccinated | 8640 | 59.9 (228/3804) | 1 (ref) |
| Measles vaccinated | 22285 | 44.9 (441/9811) | 0.84 (0.68-1.02) |
| Early age (<274 days) | 5740 | 41.9 (106/2528) | 0.75 (0.58-0.97) |
| Recommended age (274-365 days) | 9706 | 44.3 (188/4245) | 0.85 (0.68-1.07) |
| Late (366-735 days) | 6839 | 48.4 (147/3038) | 0.95 (0.73-1.23) |
| **Boys** |  |  |  |
| Measles unvaccinated | 4368 | 59.8 (115/1922) | 1 (ref) |
| Measles vaccinated | 11345 | 46.2 (231/4995) | 0.85 (0.66-1.10) |
| Early age (<274 days) | 2936 | 48.1 (62/1290) | 0.86 (0.62-1.19) |
| Recommended age (274-365 days) | 4959 | 42.8 (93/2170) | 0.81 (0.60-1.10) |
| Late (366-735 days) | 3450 | 49.5 (76/1535) | 0.95 (0.68-1.33) |
| **Girls** |  |  |  |
| Measles unvaccinated | 4272 | 60 (113/1883) | 1 (ref) |
| Measles vaccinated | 10940 | 43.6 (210/4816) | 0.82 (0.63-1.07) |
| Early age (<274 days) | 2804 | 35.5 (44/1238) | 0.65 (0.45-0.93) |
| Recommended age (274-365 days) | 4747 | 45.8 (95/2075) | 0.89 (0.66-1.20) |
| Late (366-735 days) | 3389 | 47.2 (71/1503) | 0.94 (0.67-1.31) |

1: Age as underlying time scale, stratified by village cluster

Abbreviations: MR: Mortality Rate; PYRS: Person years; HR: Hazard Ratio; CI: Confidence interval
